# Genomic Surveillance of *Salmonella* Paratyphi A: Neglected No More?

**DOI:** 10.1093/ofid/ofad077

**Published:** 2023-06-02

**Authors:** Yogesh Hooda, Arif Mohammad Tanmoy, Samir K Saha, Senjuti Saha

**Affiliations:** Child Health Research Foundation, Dhaka, Bangladesh; Child Health Research Foundation, Dhaka, Bangladesh; Child Health Research Foundation, Dhaka, Bangladesh; Department of Microbiology, Bangladesh Shishu Hospital and Institute, Dhaka, Bangladesh; Child Health Research Foundation, Dhaka, Bangladesh

**Keywords:** antimicrobial resistance, bacterial vaccines, enteric fever, genomic surveillance, *Salmonella* Paratyphi A

## Abstract

*Salmonella enterica* serovar Paratyphi A, the causative agent of paratyphoid fever, is a neglected tropical disease with a high burden and mortality in low- and middle-income countries. Limited information is available regarding its genomic diversity, especially from South Asian countries that are collectively responsible for >80% of all paratyphoid cases. At the 2021 International Conference on Typhoid and Other Salmonelloses, researchers from the around the globe presented their work on *Salmonella* Paratyphi A genomics. Presentations described recent genomic data from South Asia and the development of *Paratype*, an open-access single-nucleotide polymorphism–based genotyping scheme, to segregate *Salmonella* Paratyphi A genomes in a systematic and sustainable manner. In this review, we attempt to summarize the progress made thus far on *Salmonella* Paratyphi A genomics and discuss the questions that remain to better understand the pathogen and develop interventions to fight it.

## Teaser

More than 1000 *Salmonella* Paratyphi A isolates have been sequenced from South Asia in the past 2 years. An open-access tool, Paratype, has been developed to track genotypes and antimicrobial resistance. However, genomic data are still limited from sub-Saharan Africa, the Middle East, and South America.

*Salmonella enterica* serovar Paratyphi A, transmitted through contaminated food and water, is the primary causative agent of paratyphoid fever. With the expansion of antibiotic therapy and improvements in water, sanitation, and hygiene, there has been a 41% decline in the overall global burden of paratyphoid fever from 1990 to 2017 [1]. However, the number remains disproportionately high in low- and middle-income countries (LMICs). In 2017, *Salmonella* Paratyphi A was estimated to have infected 3.4 million people globally, causing >19 000 deaths [1]. Almost all of these cases occurred in LMICs: 2.8 million cases (82%) occurred in South Asia, 0.34 million cases (10%) in Southeast/East Asia, and 0.22 million cases (6%) in sub-Saharan Africa. Despite these high numbers, paratyphoid remains a neglected tropical disease [1].

The neglect of paratyphoid fever has likely stemmed from multiple factors. First, it is symptomatically similar to typhoid fever (caused by *Salmonella enterica* serovar Typhi) and hence, most of its clinical and treatment strategies are based on typhoid fever. Second, barring a few regions, paratyphoid is less prevalent than typhoid fever [1]. Third, unlike typhoid fever, there are no licensed vaccines for paratyphoid fever. Finally, paratyphoid infections primarily occur in LMICs where local researchers often lack the research capacity to investigate diseases. Genomic sequencing and surveillance have the potential to play a role in overcoming some of the gaps in data regarding paratyphoid fever. Systematic genomic surveillance can facilitate understanding of the diversity of *Salmonella* Paratyphi A circulating globally and investigating its transmission routes thereby allowing effective design of pharmaceutical and nonpharmaceutical interventions to further reduce the burden posed by this pathogen.

##  

### *Salmonella* Paratyphi A Genomics: Current Status

**Figure 1.**
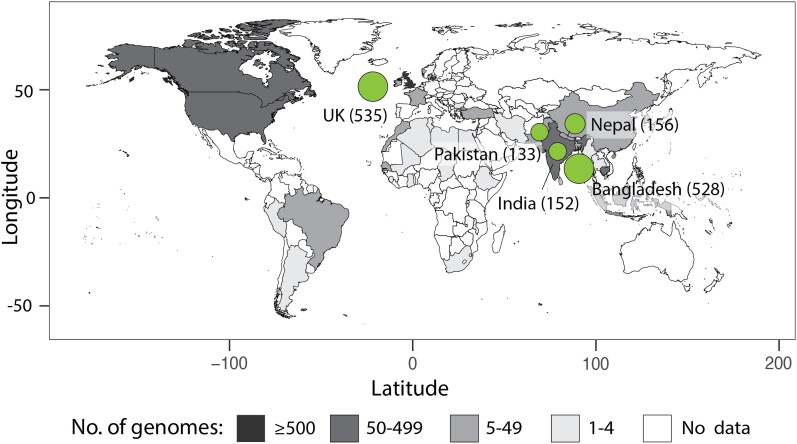
Global distribution of *Salmonella* Paratyphi A genomic data. Countries are shaded based on the number of *Salmonella* Paratyphi A genomes from the country available on the database Enterobase [12]. The genomic data presented at the 2021 International Conference on Typhoid and Other Salmonelloses are shown as green circles. Sequences were presented from the United Kingdom (535), Bangladesh (528), Nepal (156), India (152), and Pakistan (133). Abbreviation: UK, United Kingdom.

The first *Salmonella* Paratyphi A genome was published in 2004 and provided the genetic basis for many of the observed clinical symptoms. Analysis of the genome highlighted the accumulation of >150 pseudogenes and loss of >30 genes found in other *Salmonella* serovars, resulting in its restriction to humans [2, 3]. In 2014, a study sequenced 149 genomes to understand the evolution of *Salmonella* Paratyphi A and estimated that the last common ancestor for this serovar emerged in the 1500s [4]. The authors also proposed a lineage scheme to track the spread of the pathogen. This lineage system was further extended by subsequent studies that have looked at Paratyphi A genomes from outbreaks in Cambodia [5] and China [6] and travel-related cases in the United Kingdom (UK) [7, 8]. Most of the cases in the UK were related to travelers from South Asia, which is unsurprising given that these countries bear the highest burden of paratyphoid fever. Between 2014 and 2021, only 3 studies had been published on genomes of *Salmonella* Paratyphi A from South Asia, first in 2018 from Nepal [9] and the next 2 in 2021 from Bangladesh [10, 11]. As of August 2022, 1885 Paratyphi A genomes have been submitted to the National Center for Biotechnology Information and 2186 to Enterobase (Figure 1, Supplementary Table 1) [12]. These studies have begun providing a deeper insight into the field of Paratyphi A genomics, but much remains to be addressed.

At the 2021 International Conference on Typhoid and Other Salmonelloses, researchers from around the globe gathered virtually to discuss the status of typhoid, paratyphoid, and other related diseases. Of the 86 oral presentations, 3 included data on *Salmonella* Paratyphi A genomics: Dr Marie Chattaway from Public Health England presented “Phylogenomics and antimicrobial resistance of *Salmonella* Typhi and Paratyphi A, B and C in England, 2016–2019”; Dr Agila Kumari Pragasam from Christian Medical College, Vellore, India, presented “Genomic analysis unveils the role of genetic changes in the emergence and persistence of *S.* Paratyphi A lineages,” and Mr Arif Mohammad Tanmoy from the Child Health Research Foundation, Dhaka, Bangladesh, presented “*Paratype*: A genomic surveillance framework and an open-source tool for *Salmonella* Paratyphi A.” In this review, we summarize the findings from these presentations and contextualize them against the existing genomic data. We also discuss future areas of investigation and open questions regarding *Salmonella* Paratyphi A that should be addressed through genomics.

### Increasing Data From South Asia

One of the most important trends have been the number of whole genome sequences of *Salmonella* Paratyphi A from South Asia. Dr Marie Chattaway described the genomic analysis of 535 *Salmonella* Paratyphi A genomic sequences obtained from patients in the UK [13]. These findings showed that 93% of the paratyphoid cases reported travel outside the UK, most commonly to South Asia. Public Health England sequences all typhoidal *Salmonella* isolates in the UK and these studies have had a large impact on our understanding of the *Salmonella* Paratyphi A genomic diversity. Additionally, Dr Agila Pragasam presented work on the analysis of 152 Paratyphi A from India, the country with the highest burden of paratyphoid fever [1]. The study represented data from 2017 to 2020 and 4 different sites in India and described the diversity of *Salmonella* Paratyphi A isolates [14]. Last, Mr Arif Mohammad Tanmoy presented sequences of 817 Paratyphi A genomes from Bangladesh, Pakistan, and Nepal [15]. This was the largest case series sequenced so far, and included isolates collected from the Surveillance of Enteric Fever in South Asia Project (SEAP).

### Standardization of Genotyping Scheme

A major limitation in the genomic characterization and analyses of *Salmonella* Paratyphi A genomes had been the lack of a standard genotyping scheme. As *Salmonella* Paratyphi A genome has a low mutation rate (1.94 × 10^−7^ single-nucleotide polymorphisms [SNPs] per nucleotide, <1 SNP per genome per year [4]), it was theoretically possible to design an SNP-based system for genotyping Paratyphi A isolates. A similar system is available for *Salmonella* Typhi, Genotyphi (https://github.com/katholt/genotyphi), that has had an extraordinary impact on tracking the geographical and temporal spread of *Salmonella* Typhi [16, 17]. Taking this into consideration, our group in Bangladesh developed a SNP-based genotyping scheme for *Salmonella* Paratyphi A, termed *Paratype*, based on 1379 Paratyphi A genomes sequenced thus far [15]. In this scheme, all *Salmonella* Paratyphi A genomes have been segregated into 3 primary clades, 8 secondary clades, and a total of 18 genotypes.

To make such genomic analysis accessible for all, a script for the *Paratype* scheme has been developed as well, which works for different file formats including fasta, fastq, bam, and vcf [15]. This script has been tested and validated on data generated by both the Illumina and the Oxford Nanopore platforms. *Paratype*, openly available through GitHub (https://github.com/CHRF-Genomics/Paratype), will facilitate genomic epidemiological studies as it provides the ability for quick analysis, without time-consuming and expertise-intensive steps like construction of an RAxML or phylogenetic tree for each analysis.

### Antimicrobial Resistance

All 3 oral presentations also investigated antimicrobial resistance of *Salmonella* Paratyphi A. Unlike in *Salmonella* Typhi, the detection of multidrug resistance (defined by resistance to ampicillin, cotrimoxazole, and chloramphenicol) in *Salmonella* Paratyphi A remains low. Furthermore, except for 1 case [18], ceftriaxone resistance is also absent. Resistance markers for multidrug resistance and ceftriaxone are usually present on plasmids, which seem to be present at a lower level in *Salmonella* Paratyphi A in comparison to *Salmonella* Typhi, suggesting the presence of an underlying mechanism that prevents acquisition or retention of plasmids in *Salmonella* Paratyphi A [15]. However, no such mechanism has been identified to date.

On the other hand, resistance against ciprofloxacin and azithromycin, which are generally mediated by single pointmutations, is found at levels similar to *Salmonella* Typhi. More than 80% of the *Salmonella* Paratyphi A genomes reported by the 3 studies were nonsusceptible to ciprofloxacin [13–15]. Ciprofloxacin resistance, caused by mutations in the quinolone resistance–determining region, were first identified in the 1990s; they were detected in >95% of isolates identified in South Asia over the last 5 years [13–15]. Azithromycin resistance is much less frequent, with only 6 azithromycin-resistant isolates found since its first detection in 2016, all from Bangladesh [10, 19].

### Future Outlook

While a tremendous growth of sequencing data from endemic countries was noted, sequencing data are still limited from many countries that have a high burden for *Salmonella* Paratyphi A. For instance, countries in sub-Saharan Africa are estimated to bear approximately 7% of global paratyphoid cases [1] but have contributed to 23 of 2186 (1%) of the genomes that are currently available on Enterobase (Supplementary Table 1). In the case of *Salmonella* Typhi, South Asia was the probable epicenter for the spread of the pathogen globally [16]. *Salmonella* Paratyphi A might also follow a similar pattern, but details remain unknown due to the gaps in sequencing data from different geographical locations. Furthermore, with factors associated with globalization, like climate change and increasing travel, paratyphoid outbreaks may become more common and spread rapidly. In 2011, the outbreak of paratyphoid fever caused by *Salmonella* Paratyphi A in the Yuegcheng district of China [6] was closely related to isolates from Pakistan, indicating the probable source of transmission. Increasing local capacity for conducting whole genome sequencing and bioinformatic analysis on site is crucial to close the data gaps and track transmission and outbreaks.

The recently developed genotyping scheme, *Paratype*, will also make it possible to identify potential sources of outbreaks in regions where paratyphoid fever is not endemic. However, it is important to sequence genomes from areas where paratyphoid is endemic, and continuously update the Paratype scheme if gaps are identified. In a positive sign for the field, the work from India presented during the conference (described above) was later reanalyzed by the authors using *Paratype* [14]. This not only led to a more granular interpretation of their results [14], but the authors were also able to identify 3 additional genotypes that were circulating in India, namely, 2.3,8, 2.3.9, and 2.4.5. Because the *Paratype* scheme is open-access, changes to the script can be made rapidly, and a new scheme can be updated by our group as per the needs of the field [15]. We aim to keep track of user notifications on GitHub about new genomes for whom genotypes cannot be predicted by *Paratype*. If >10 such genomes are obtained, we will generate a phylogenetic RAxML tree and check for the presence of new clusters. If a new cluster is indeed identified, we will identify the unique alleles for the cluster and assign a new genotype.

**Figure 2.**
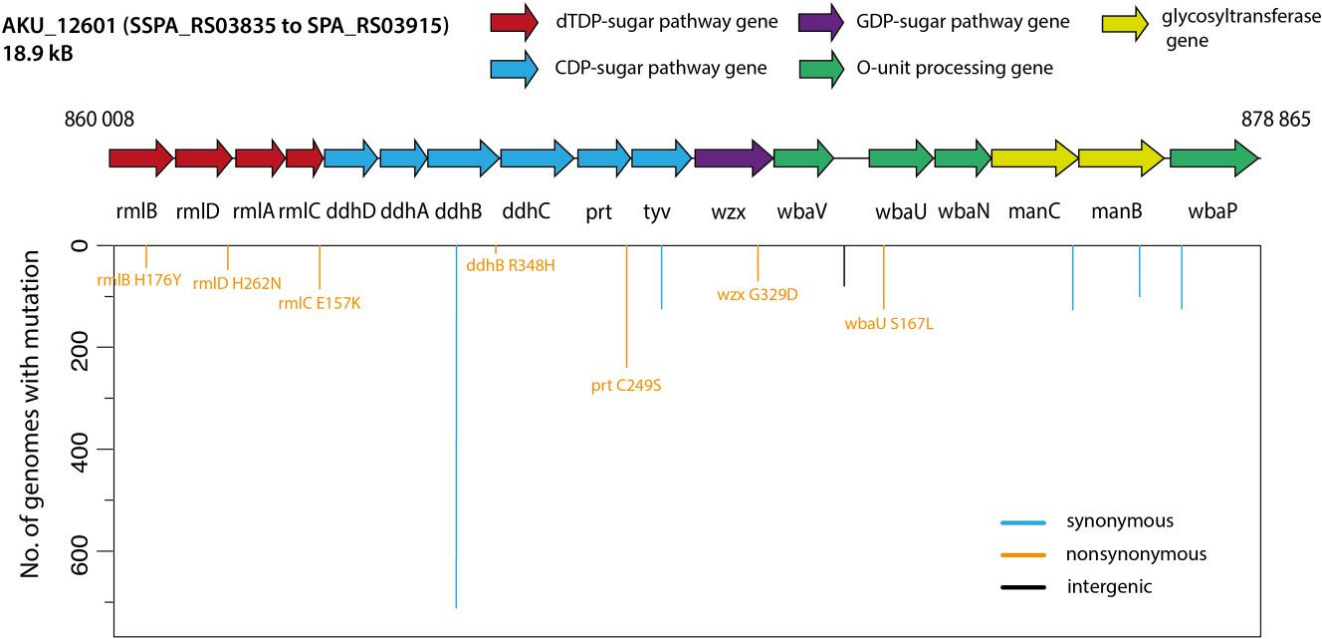
Single-nucleotide mutations in the O2-specific region in *Salmonella* Paratyphi A. The 13 mutations in the O2-specific region in *Salmonella* Paratyphi A found in >10 isolates are mapped (reference accession: FM200053.1) with synonymous, nonsynonymous, and intergenic single-nucleotide polymorphisms highlighted. The resulting amino acid changes due to nonsynonymous mutations are labeled. Abbreviations: CDP, cytidine diphosphate; dTDP, deoxythymidine diphosphate; GDP, guanosine diphosphate.

Another area of future investigation would be identification of vaccine antigens for *Salmonella* Paratyphi A. Several vaccines are currently under investigation and the most common vaccine target is the O2-antigen, which is unique to *Salmonella* Paratyphi A and not found on other *Salmonella* species [20–22]. O2-antigen biosynthesis requires 17 genes that are encoded on a single 18.9-kb locus on the *Salmonella* Paratyphi A genome (Figure 2) [23]. We identified the SNPs in the O2-antigen biosynthesis genes found in the 1379 genomes. In total, 84 SNPs were found, of which 13 were present in >10 genomes. The most common SNP was at the genomic location 868 444 of the reference accession, FM200053.1 (G > C; synonymous mutation in *prt* gene encoding paratose synthase) and was found in 17% (239/1379) of all isolates. Of these 13 common SNPs, 7 led to nonsynonymous mutations (Figure 2) that could potentially change the O2-antigen structure and chemistry. However, there are no data clarifying if any of these mutations influence the chemistry of the O2-antigen or antibodies elicited upon vaccination. Furthermore, no data are available on the role of O2 antigen in pathogenesis. As the O2-antigen proceed in the clinical trials, these questions will be important to judge the efficacy of the vaccine candidates and track their rollout. Such monitoring can only be conducted using regular whole genome sequencing in endemic countries, in real time.

The progress that has been made on *Salmonella* Paratyphi A biology and epidemiology in recent years portends positive signs in the field of genomics and global health. Paratyphoid fever is endemic in many LMICs and 2 of the studies discussed here were largely conducted in their respective countries. The development of *Paratype*, a genotyping scheme from Bangladesh, also represents one of the first examples of bioinformatic tools developed by scientists in LMICs and would be useful for the public health community across the Global North and Global South. Such efforts must be nurtured if the decentralization of genomics and decolonization of the global health agenda are to be achieved. An area that requires immediate attention is the paucity of bioinformatics skills among researchers in LMICs that prevents them from analyzing their own data. Moreover, development of bioinformatics tools is often supported by unrestricted grants to research groups at universities as funding agencies in global health often focus on primary data generation. However, tools such as *Paratype* have the potential to empower researchers across countries and require dedicated funding streams to ensure that such tools are regularly maintained and updated. We must address these gaps if we want to make global health truly global and remove the neglect from neglected tropical diseases.

## Supplementary Data

Supplementary materials are available at *Open Forum Infectious Diseases* online. Consisting of data provided by the authors to benefit the reader, the posted materials are not copyedited and are the sole responsibility of the authors, so questions or comments should be addressed to the corresponding author.

## Supplementary Material

ofad077_Supplementary_Data

## Notes

***Author contributions.*** Y. H. and S. S. wrote the first draft, made the figures, and compiled the final manuscript. A. M. T. and S. K. S. provided critical comments and edited the documents. All authors agreed to the final draft of the manuscript.

***Acknowledgments.*** We thank members of the Clinical Microbiology and Genomics teams at Child Health Research Foundation for their discussions and comments. We thank Ms Nazifa Tabassum for proofreading the draft and providing editorial comments.

***Supplement sponsorship.*** This article appears as part of the supplement “Charting the Course to Meet the Challenges Ahead: Research and Developments on Typhoid and Other Invasive Salmonelloses” sponsored by the Coalition against Typhoid Secretariat, housed at the Sabin Vaccine Institute in Washington, DC and made possible by a grant from the Bill & Melinda Gates Foundation.
