# Supplementary material for: Genomic Surveillance of Salmonella Paratyphi A: Neglected No More?
Source: Open Forum Infect Dis. 2023 Jun 2;10(Suppl 1):S53–7. doi: 10.1093/ofid/ofad077 (PMC10236518; doi:10.1093/ofid/ofad077)
Supplement: ofad077_Supplementary_Data [file ofad077_supplementary_data.docx]

**Supplementary Data:**

**Supplementary Table 1:** Country breakdown of sequenced isolates of *Salmonella* Paratyphi A at Enterobase

| **Country** | **Count** | **Time span** |
| --- | --- | --- |
| Albania | 1 | 1917 |
| Algeria | 3 | 1949-1962 |
| Argentina | 2 | 2017 |
| Bangladesh | 5 | 2001-2015 |
| Belgium | 4 | 2001-2014 |
| Brazil | 5 | 1960-2008 |
| Bulgaria | 1 | 2004 |
| Cambodia | 147 | 1958-2015 |
| Canada | 109 | 1995-2018 |
| Chad | 1 | 2000 |
| China | 22 | 1955-2019 |
| Denmark | 5 | 1928-2001 |
| Egypt | 1 | 1954 |
| Ethiopia | 1 | 1956 |
| France | 12 | 1945-2002 |
| Gambia | 1 | 2007 |
| Ghana | 1 | 2007 |
| Guinea | 3 | 2000-2002 |
| Hong Kong | 2 | 1971 |
| India | 107 | 1943-2019 |
| Indonesia | 7 | 1955-2010 |
| Iran | 1 | 1949 |
| Ireland | 15 | 2016-2020 |
| Israel | 2 | 1925 |
| Jordan | 1 | 1934 |
| Korea | 1 | NA |
| Kuwait | 2 | 2006 |
| Luxembourg | 1 | 2014 |
| Malawi | 1 | 2005 |
| Mali | 3 | 1977-2006 |
| Morocco | 5 | 1957-2000 |
| Myanmar [Burma] | 2 | 2003-2005 |
| Nepal | 14 | 1999-2009 |
| Pakistan | 15 | 1997-2021 |
| Peru | 4 | 2001-2010 |
| Senegal | 5 | 1952-2006 |
| Sierra Leone | 1 | 2008 |
| Singapore | 1 | 2016 |
| South Africa | 4 | 2021-2022 |
| Sri Lanka | 1 | 1999 |
| Sudan | 1 | 2005 |
| Thailand | 1 | 1999 |
| Tunisia | 1 | 1954 |
| Turkey | 5 | 1952-2004 |
| United Kingdom | 1108 | 2004-2021 |
| United States | 118 | 1899-2021 |
| Vietnam | 3 | 1946-2005 |
| Zambia | 1 | 2018 |
| Not known | 429 | NA |
| **Total** | **2186** | **1899-2021** |
